# Immunity and Genetics at the Revolving Doors of Diagnostics in Primary Immunodeficiencies

**DOI:** 10.3390/diagnostics11030532

**Published:** 2021-03-16

**Authors:** Francesco Rispoli, Erica Valencic, Martina Girardelli, Alessia Pin, Alessandra Tesser, Elisa Piscianz, Valentina Boz, Flavio Faletra, Giovanni Maria Severini, Andrea Taddio, Alberto Tommasini

**Affiliations:** 1 Department of Medical, Surgical and Health Sciences, University of Trieste, 34149 Trieste, Italy; francesco.rispoli@outlook.com (F.R.); valentina.boz@burlo.trieste.it (V.B.); andrea.taddio@burlo.trieste.it (A.T.); alberto.tommasini@burlo.trieste.it (A.T.); 2 Department of Pediatrics, Institute for Maternal and Child Health—IRCCS “Burlo Garofolo”, 34137 Trieste, Italy; martina.girardelli@burlo.trieste.it (M.G.); alessia.pin@burlo.trieste.it (A.P.); alessandra.tesser@burlo.trieste.it (A.T.); elisa.piscianz@burlo.trieste.it (E.P.); giovannimaria.severini@burlo.trieste.it (G.M.S.); 3 Department of Diagnostics, Institute for Maternal and Child Health—IRCCS “Burlo Garofolo”, 34137 Trieste, Italy; flavio.faletra@burlo.trieste.it

**Keywords:** primary immunodeficiencies, recent thymic emigrants, X-chromosome inactivation, autoinflammatory diseases, lymphoproliferative immune defects, mendelian susceptibility to infections, flow cytometry, next generation sequencing

## Abstract

Primary immunodeficiencies (PIDs) are a large and growing group of disorders commonly associated with recurrent infections. However, nowadays, we know that PIDs often carry with them consequences related to organ or hematologic autoimmunity, autoinflammation, and lymphoproliferation in addition to simple susceptibility to pathogens. Alongside this conceptual development, there has been technical advancement, given by the new but already established diagnostic possibilities offered by new genetic testing (e.g., next-generation sequencing). Nevertheless, there is also the need to understand the large number of gene variants detected with these powerful methods. That means advancing beyond genetic results and resorting to the clinical phenotype and to immunological or alternative molecular tests that allow us to prove the causative role of a genetic variant of uncertain significance and/or better define the underlying pathophysiological mechanism. Furthermore, because of the rapid availability of results, laboratory immunoassays are still critical to diagnosing many PIDs, even in screening settings. Fundamental is the integration between different specialties and the development of multidisciplinary and flexible diagnostic workflows. This paper aims to tell these evolving aspects of immunodeficiencies, which are summarized in five key messages, through introducing and exemplifying five clinical cases, focusing on diseases that could benefit targeted therapy.

## 1. Introduction

According to an old paradigm, the possibility of a primary immunodeficiency disease (PID) should be considered in subjects with recurrence of serious infections. Whilst the easy assessment of blood cell count and immunoglobulin levels together with a careful evaluation of clinical data can address the suspicion of PID in many severe cases, the availability of specific immunological phenotyping in the last twenty years of the 20th century led to a significant advance in the diagnosing of this group of disorders [1]. Flow cytometry-based classification also facilitated studies leading to the identification of many genetic errors underlying PIDs. Even after the identification of causative genes for several defects, laboratory immune evaluation remained crucial for diagnosis of many PIDs due to the prompt availability of the results and to the functional significance of assays that could reflect the severity of the underlying molecular defect.

More recently, the widespread use of molecular genetics greatly increased the capacity to identify PIDs, and correlations between genotypes and phenotypes have begun to be established, raising the question whether it was still justified to base a diagnosis only on a clinical and phenotypical evaluation [2,3].

With improved capability of diagnosing immune defects, a new paradigm of PID emerged, showing that immunodeficiencies can be characterized by autoimmune, lymphoproliferative, and neoplastic symptoms, in addition to infections [4]. Indeed, a search for PID is now recommended in subjects with refractory cytopenia or with multiple autoimmune manifestations. Furthermore, even a single severe infection, especially if it arises from a commensal or unusual pathogen, can raise the suspicion of a PID. Thus, according to a new paradigm, a PID should be considered in subjects with a wide range of infectious, inflammatory, and autoimmune manifestation due to an improper immune function. Even if cytometry laboratories of immunology have evolved improved panels to take into account a wider range of phenotypes [5], immunophenotyping can yield normal results in many cases, and thus, it cannot be used as the sole tool to rule out the diagnosis of a PID, with maybe the exception of Severe Combined Immunodeficiencies (SCIDs).

Conversely, improved molecular diagnostics allowed us to search for disease-causing mutations in hundreds of immune-related genes simultaneously by exploiting next-generation sequencing (NGS) capabilities. Gene panels have been implemented for distinct groups of PIDs and, more recently, clinical exome sequencing has been proposed to analyze hundreds of PID genes at once. Current guidelines recommend moving on from targeted gene panels to clinical exome data, which can be analyzed starting from nearly 400 genetic defects identified in patients with distinct primary immunodeficiency [6]. In the cases in which there is no obvious genetic explanation for the disease, the analysis may be further expanded in silico. The use of targeted gene panels can still be applied in cases in which the clinical suspicion is oriented toward a narrow set of genes, allowing higher sequences coverage and an easier first-line evaluation of results. In the selected case, whole genome sequencing can be preferred due to the possibility of identifying copy number variations, large deletions, and other genomic rearrangements [7], as reported by some authors [8,9,10], even though it requires complicated analysis given the large amount of data and thus should be performed as a second-line investigation. Overall, exome and genome sequencing give the opportunity to re-analyze data in the light of newly discovered disease-related genes in patients without a genetic diagnosis. However, several pitfalls can hinder the interpretation of genetic results. First, exome sequencing may miss structural defects such as gene inversions or large deletions, and some genes may be poorly covered in NGS, as in the example of *IKBKG* due to the presence of a closely related pseudogene. Moreover, disease-causing mutations present in somatic mosaicism in a proportion of cells can be hardly identified by conventional techniques, as in the case of adult-onset autoimmune lymphoproliferative syndrome. Thus, the appropriate genetic testing should be thoroughly considered based on the strength of clinical suspicion of a given condition. Lastly, genetic studies may yield lots of data of uncertain significance, requiring new immunologic studies for a definite confirmation.

We present case studies to discuss how the diagnostics of PIDs moved from a straightforward sequence, which led from the clinical picture to hematological and flow cytometry diagnostics, to a more articulated multidisciplinary workflow connecting various clinical specialties and requiring the collaboration between laboratory immunologists and geneticists. Of note, in some cases, the knowledge of molecular defects has allowed targeting the diseased mechanism with precision drugs [11,12,13]. This is especially true for PIDs associated with Gain-of-Function pathways [14] that can be targeted by pharmacological inhibitors, which could be already available as developed to cure cancers [15]. The early detection of such “druggable” disorders is crucial to prevent the development of severe organ damage [16].

By exploiting the discussion of a case series, we present five typical scenarios, highlighting some developments in diagnostics of PIDs. This article could scarcely describe the complex field of PIDs and is not intended to give diagnostic recommendations. It is just meant to discuss how the diagnostics of PIDs is moving in the era of NGS, requiring an intimate collaboration among various specialists in multidisciplinary teams. Some tables are provided to highlight how some key assays such as the measure of recent thymic emigrants (RTE) or the assessment of X-chromosome inactivation (XCI) could contribute together with genetic analysis to the diagnosis of PIDs. Particular attention is paid to disorders that can be effectively treated by hematopoietic stem cell transplantation, gene therapy, or molecularly targeted therapies.

## 2. Case Presentation

### 2.1. Case 1. Recent Thymic Emigrants

Case #1 was a firstborn male born at full term after a normal pregnancy without perinatal problems. At the start of weaning, around five months of age, his weight started to grow less. After some months of poor weight growth, he performed a wide range of blood tests that showed normal complete blood count (CBC), hepatic and renal function indices, electrolytes, and no antibodies for celiac disease. The only pathological finding was agammaglobulinemia. The infant had no infections until nine months of age when he was admitted to intensive care with respiratory failure due to interstitial pneumonia caused by Pneumocystis jirovecii. Among the investigations for PID, a flow cytometric study of lymphocyte subsets was diagnostic of T-B+ NK-SCID (supplementary materials). Male sex, the period of onset and clinical features, along with typical immunophenotype, were suggestive of X-linked SCID (X-SCID). As expected, genetic tests revealed an *IL2RG* gene mutation (c.741insG:p.S248fs) [17], confirming the clinical suspicion. At the age of 11 months, the patient received a bone marrow transplant with good outcome and no significant complications.

### 2.2. Case 2. X-Linked PIDs

A male baby was born by spontaneous delivery after a full-term pregnancy complicated by intrauterine growth restriction. Given his low birth weight, he was admitted to the neonatal unit for clinical observation and initiation of early feeding. At approximately three hours of life, the neonate manifested diffuse petechiae, predominantly in the trunk and back, and a left parietal cephalohematoma. Investigations for bleeding showed normal coagulation function but severe isolated thrombocytopenia (13,000/mmc, MPV 8.2 fL). Bone marrow aspirate was normocellular and showed no blastic cells, rare megakaryocytes of variable dysmorphic appearance, and no signs of dyserythropoiesis. Familial history was contributive for a maternal uncle death at two years of age from cerebral hemorrhage due to an undefined congenital platelet disease (genetic investigations were not available at the time), and a great-uncle death at four months of age from sepsis. In the first days of life, the infant developed fever, general decay, and jaundice, so he started empirical antibiotics and phototherapy. Due to the persistence of unexplained jaundice after microbiological and radiologic examination, a liver biopsy was performed, which showed a histologic picture compatible with either herpes virus hemophagocytic syndrome or maternal GVHD (graft-versus-host disease), which was supportive of an underlying immunodeficiency. Flow cytometry analysis performed in the suspicion of Wiskott–Aldrich syndrome (WAS) showed absent WASP expression (supplementary materials). Directed sequencing of the *WAS* gene on the X-chromosome led to the detection of a likely pathogenic mutation (c.708delT:p.A236AfsX24), which has never been described before (supplementary materials). The evidence of a skewed XCI pattern in the mother’s peripheral blood cells (supplementary materials), together with the pedigree compatible with X-linked disorders, eventually confirmed the pathogenic role of the mutation. Unfortunately, the boy died from transplant-related complication with severe infections.

### 2.3. Case 3. Immunodeficiencies with Autoinflammation

A 7-year-old girl came to our attention for hepatopathy with positive antinuclear antibodies (ANA) but with normal results from liver biopsy. She also complained of recurrent aphthous stomatitis since the age of four years, with some episodes complicated by oral thrush and painful swallowing. She had been previously evaluated with the suspicion of Behçet disease, but without reaching a definite diagnosis. In the last years, she also presented a couple of episodes of paronychia, which had been considered due to the girl’s habit of biting the skin of her fingers. When she referred to our hospital, laboratory investigations revealed increased aminotransferase, raised erythrocyte sedimentation rate (ESR), high-titer ANA, positive direct Coombs test, and positive anti-double-stranded DNA antibodies. Furthermore, a strikingly positive interferon score was measured in her peripheral blood (supplementary materials). A diagnosis of systemic lupus erythematosus (SLE) was made and a treatment with mycophenolate mofetil (MMF) and hydroxychloroquine started, together with on-demand fluconazole for oral candidiasis. Whilst both aminotransferase and ESR dropped to nearly normal levels, episodes of oral aphthosis with superimposed oral candidiasis persisted, as well as recurrent paronychia. Colchicine was added to reduce the recurrence of oral aphthosis. Furthermore, a PID was suspected, and targeted genetic testing was performed (supplementary materials), revealing c.862A>G mutation in the *STAT1* gene (p.T288A, rs387906765), which confers an increased activity of the STAT1 protein [18]. This condition, called “STAT1 Gain-of-function” (GOF) syndrome, is in fact characterized by the presence of chronic mucocutaneous candidiasis in almost all cases and autoimmune disease in a great proportion of affected patients [19]. Based on this genetic diagnosis, a treatment with a JAK 1/2 inhibitor (baricitinib, 2 mg once daily) was started instead of MMF, obtaining good control of the disease.

### 2.4. Case 4. Immunodeficiencies with Lymphoproliferation

The fourth patient is a woman with a clinical history of recurrent respiratory infections, in some cases requiring hospitalization, and a few episodes of colitis, up to 20 years of age. After a period of well-being that lasted about ten years, pulmonary and urinary infections began to recur. Immunological tests were performed that showed an IgA defect (23 mg/dL). In addition to recurring infections, she developed alopecia areata at ten years of age and eczematous lesions on knees and genitals during puberty. Over various consultations, several non-specific findings were noted: mild Diffusing capacity of the Lungs for Carbon monOxide (DLCO) reduction, dermal hyperplasia, mild gastric atrophy, mild colic eosinophilic inflammation, and a peculiar hypertrophy of the lingual tonsil. Further laboratory investigations revealed a mild lymphopenia (960/mcrL) with B-cell defect. The woman was referred to our center by an Ear Nose and Throat specialist because of the uncommon finding of a lingual tonsil hypertrophy. Immunophenotyping evidenced an inversion of the CD4/CD8 ratio and increase of senescent (CD57 + CD45RA+) CD8 T cells, which, together with low IgA values, supported the hypothesis of a primary immunodeficiency (supplementary materials). Furthermore, lingual tonsil hypertrophy was noticed as a sign of abnormal lymphoproliferation. A genetic panel for PID predominantly associated with hypogammaglobulinemia evidenced the presence of the variant c.G454A:p.A152T in the *PIK3CD* gene (rs138463758) (supplementary materials), which was consistent with a possible diagnosis of activated phosphoinositide 3-kinase δ syndrome (APDS). Again, we were faced with a variants of uncertain significance (VUS), the pathogenicity of which had to be confirmed. Knowing the specific molecular pathway to which the mutated gene’s protein encoded belongs, it is possible to use ancillary tests to evaluate the pathway’s expression of molecules. In our case, the mutation was in PIK3CD, which is a molecule of the PI3K/AKT/mTOR/S6K pathway, so a test was performed to evaluate the functionality of the cascade, namely the phosphorylation of the downstream molecule S6 in B cells, which proved to be high (S6 more phosphorylated) [20] and therefore indicated that there was increased PIK3CD activity: that means GOF mutation. Of note, IgA deficiency, increase of senescent CD8 and lymphoproliferative features are all typical features of APDS in addition to recurrent infections [21]. A therapeutic attempt with theophylline 200 mg daily was attempted for six months, based on a previous experience in a younger case with type II APDS (due to mutations in *PIK3R1* [22]), however without a clear benefit. The opportunity of starting a treatment with leniolisib, a specific inhibitor of PIK3CD, is currently under evaluation.

### 2.5. Case 5. Susceptibility to: EBV, Mycobacteria, Candida, Warts

Case #5 is about an 18-year-old boy patient admitted for mononucleosis with pulmonary, hepatic, and cerebral involvement with seizures. Polymerase chain reaction (PCR) examination on blood and bronchoalveolar lavage demonstrated the presence of high viral load Epstein–Barr virus (EBV). At the same time, signs of lymphoproliferation appeared (systemic lymphadenomegaly, hepato-splenomegaly with poor liver function indices, elevated serum lactate dehydrogenase). A worsening of respiratory function followed, with the need for intubation and transfer to ICU. Based on the diagnosis of complicated EBV infection, a treatment was started with antiviral and rituximab. After initial improvement in respiratory function and extubation, the patient had a relapse that affected the nervous system and led to a state of coma with the need for a second intubation. EBV DNA was amplified by PCR in cerebrospinal fluid, while blood examination showed moderate-to-severe anemia, hypofibrinogenemia, and hypertriglyceridemia. Overall, these data were consistent with a diagnosis of severe EBV infection complicated with hemophagocytic lymphohistiocytosis, leading to the search of genetic susceptibility to this kind of infection. Genetic analysis revealed that the boy was hemizygous for the c.3G>C p.M1I mutation in the *SH2D1A* gene, allowing the diagnosis of X-linked lymphoproliferative disorder (XLP). Based on these results, the patient underwent therapeutic conditioning followed by bone marrow transplantation, with good long-term outcome.

## 3. Discussion

**Figure 1:**
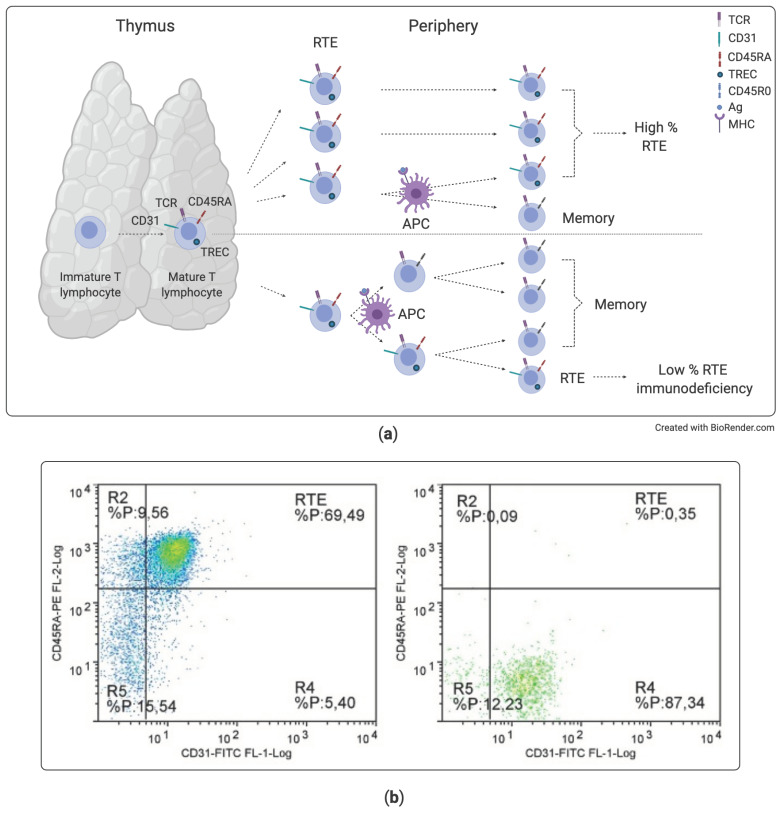
Recent thymic emigrants (RTE) in healthy people and in subjects with immunodeficiencies arising from defective TCR recombination. (**a**) Difference in the portion of RTE lymphocytes between normal and immunodeficiency condition, at equal lymphocyte count. A subject with immunodeficiency may present a smaller fraction of thymic-matured T cells, which do not yet expand in the periphery after antigen (Ag) encounter. APC: antigen-presenting cell. MHC: major histocompatibility complex. (**b**) Analysis of RTE in a healthy control (left dot plot) and in a patient affected with X-linked SCID (X-SCID) (right dot plot). The cytograms are obtained after gating on CD45^high^, CD3+, and CD4+ population. RTEs are identified by the co-expression of CD45RA and CD31 on CD4 T cells (upper right quadrant).

Case #1 was a typical case of SCID. The description of the case serves to illustrate the importance of reliable methods to measure thymic output both for diagnosis and in newborn screening. SCIDs are the most severe PIDs, presenting in the first year of life with various combinations of symptoms, ranging from severe infections, often from opportunist germs, to inflammatory and autoimmune manifestation, such as enteropathy, dermatitis, and autoimmune cytopenia. An SCID can be suspected also in infants with unexplained failure to thrive or with slow healing from a trivial infection. Infants with SCID usually have low lymphocyte count: attention should be paid to percentiles for age, since in the first months of life, reference values are much higher than ever [23]. Low levels of immunoglobulins can contribute to raised suspicion of PID. However, since the lymphocyte count may be normal in a few cases, lymphocyte immunophenotyping is necessary to confirm or rule out the diagnosis [5]. The most informative subset in evaluating an SCID is represented by RTE, which are a direct measure of the thymic output of naive cells, with a proper maturation of their T cell receptor. RTE are identified by the co-expression of CD3, CD45RA, and CD31 and are better evaluated on the subset of CD4 T cells [24,25]. The number of RTE well correlates with the results of the T cell receptor excision circle (TREC) assay, which measures the number of T cells bearing the genetic print of an effective receptor maturation [24,26,27] (Figure 1a). Whilst the RTE assay by flow cytometry is currently used for the analysis of cases with suspicion of a SCID, the TREC assay is best suited for analysis on dried blood spots in newborn screening for SCIDs [28]. Indeed, infants with a familial history of SCID who received the diagnosis of the disease at birth had better outcomes compared with their siblings in whom the diagnosis had been made because of symptoms, as they could be cured by hematopoietic stem cell transplantation in elective conditions [29]. This is one of the main reasons that fostered initiatives of SCID screening in several countries. The availability of the TREC assay, and of κ-deleting recombination excision circles (KREC) assays investigating B cell receptor recombination, made it possible to add SCID among several genetic disorders screenable on Guthrie’s card blood spots [30].

In the case described, the abnormal lymphocyte subpopulation pattern (lacking T cells and NK cells, but with normal numbers of B cells) and the low number of RTE allowed addressing the suspicion toward an X-SCID (Figure 1b), and the diagnosis was promptly confirmed by the sequencing of the gamma-chain gene. In this case, clinical and immunological data were sufficient to guide the right diagnosis.

In cases with a reference TREC assay at the screening, the diagnostic path is less straightforward: if results are confirmed in a second blood spot, a clinical and immunological evaluation must be performed promptly, especially in cases with undetectable TREC results. Immunophenotype must include the assessment of RTE [31]. Preterm newborns with low but not absent TREC can be followed for some weeks before intensive investigation for an SCID [32,33,34]. In subjects with confirmed pathological results, genetic analysis can be performed by direct gene sequencing or by NGS of several SCID-related genes at once. Immunologic investigation may be required again to improve the interpretation of results of uncertain significance obtained by NGS panels. Of note, newborns with the SCID due to adenosine deaminase deficiency (ADA-SCID) may have normal levels of TRECS, as the toxic metabolites produced because of the enzymatic deficiency are detoxified in utero through the placenta. Thus, ADA-SCID is not detected by TREC screening but by the biochemical measure of adenosine and 29-deoxyadenosine [35]. Table 1 shows a list of conditions in which the measure of RTE or TREC can be contributive for diagnosis.

The Case #2 description serves to illustrate how the analysis of X-chromosome inactivation still plays a significant role in diagnostics of PIDs. In this case, the diagnosis was straightforward, considering the typical clinical presentation and the familial history. Wiskott–Aldrich should always be considered in newborns with thrombocytopenia even if a small platelet volume can sometimes be missed by automatic counters, while it can be noticed by an experienced hematologist looking at peripheral blood smears. In our case, the diagnosis was supported by flow cytometry results showing absent WASP expression together with increased CD4/CD8 ratio. A skewed XCI pattern in the mother linked these results with the familial history of a death from infection in a maternal uncle.

XCI assay has been an important test supporting the suspicion of specific X-linked PIDs in the pre-genomic era [61,62]. Even if XCI tended to go into disuse with the advent of NGS, it still retains usefulness, both to reinforce suspicion of an X-linked PID and to indirectly support the pathogenic role of variants of uncertain significance (VUS) identified by genetic analyses.

**Figure 2:**
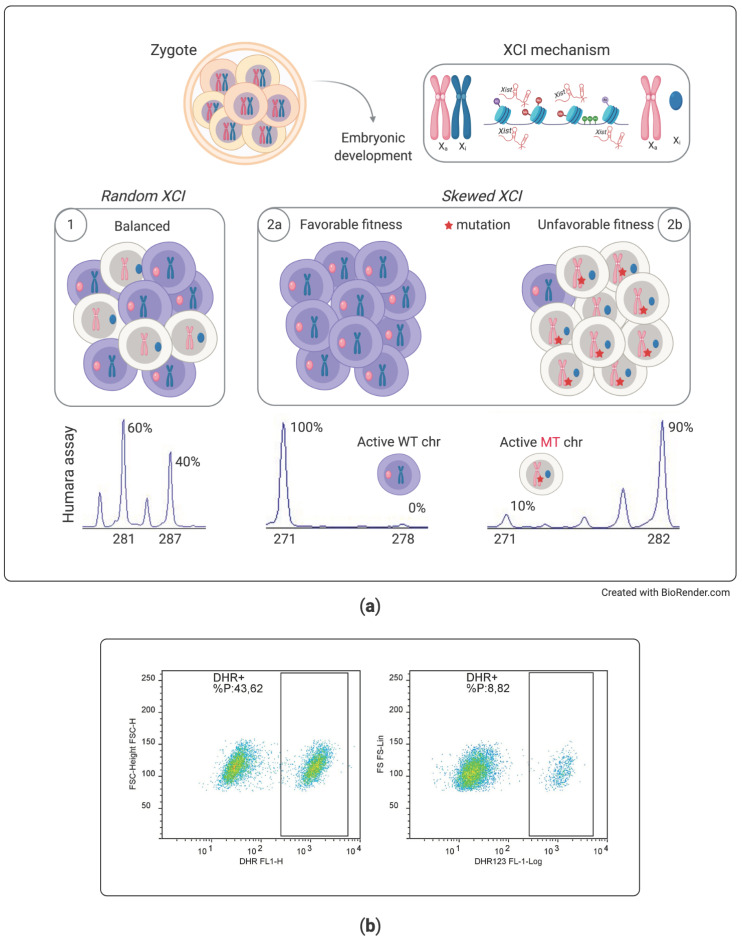
X-chromosome inactivation (XCI) assay in females heterozygous for X-recessive immune disorders. (**a**) A random X-chromosome inactivation occurs in each female during embryogenesis, and the expected inactivation percentage is estimated about 50% (1). However, nonrandom or skewed XCI may be found in peripheral blood if the female is heterozygous for a mutation affecting cell proliferation (2). For example, if a mutation is associated with defective cell maturation, only cells that activated the wild-type allele will be found in the blood of female carries, who will be completely healthy (2a). However, in some cases, one of the two X-chromosome may be silenced due to the presence of some other structural chromosomal abnormality. In these cases, the other X-chromosome will be always active, even if it carries mutation in immune genes. In these unfavorable conditions, females may express X-recessive immunodeficiency (2b). Human androgen receptor assay (HUMARA) allows us to indirectly assess the relative activation of the two X-chromosomes. The *AR* gene presents a hypervariable CAG short tandem repeat that permits distinguishing between the paternally and maternally derived X-chromosome (the peaks present in the figure above). Exploiting methylation-sensitive restriction sites to selectively digest the active allele (unmethylated) allows us to distinguish the percentage of active (enzyme digested and not amplified with PCR) from the inactive alleles. (**b**) Flow cytometric analysis of the DHR test performed in two female carriers of X-linked CGD (supplementary materials). Deficiency in the *CYBB* gene associated with CGD only impacts granulocyte function and not their proliferative fitness; thus, heterozygous females normally display random XCI and are healthy (left panel); in rare cases, XCI can be skewed for other unknown factors: heterozygous females displaying skewed XCI may have too low a percentage of functional neutrophils and can thus express the disease (right panel).

Basically, due to the random lyonization process, female cells usually display random XCI. However, a skewed pattern can be recognized if one of the two X-chromosomes bears gene mutation resulting in the reduced proliferation/survival fitness of particular types of cells [63]. For example, since functional BTK is required for the differentiation of B cells, female carriers for pathogenic BTK mutation will have only peripheral B cells expressing the wild-type allele, while staminal cells expressing the diseased BTK allele will fail to differentiate into B cells. Thus, the XCI assay will show a skewed pattern in B cells and a random one in other cell types (Figure 2) [61]. The findings from XCI in female heterozygous patients for diverse X-linked PIDs are shown in Table 2. Even if XCI analysis may be useless in cases with de novo mutation not inherited by the mother, it can be highly contributive when a skewed inactivation profile is demonstrated in the proper subset of the mother’s cells. In almost all cases, female heterozygous patients for X-linked PIDs are completely healthy, but rare exceptions may exist in subjects having a skewed XCI for other reasons (Table 2). In these cases, flow cytometry analysis can show double peaks reflecting the presence of two cell populations, activating respectively the wild-type or the diseased allele (Figure 2).

Today, NGS panels or clinical exome sequencing are increasingly used to assist the genetic diagnosis in the field of X-linked PIDs [93,94]. However, as discussed above, some X-linked PIDs are so well characterized to justify a provisional diagnosis based only on clinical and immunological data. For example, this is true for WAS, X-SCID and CGD. Of note, in some cases with high clinical suspicion of these disorders, exome-based genetic analysis can yield false negative results, and more complex genomic analyses are required. In such cases, the reliability of flow cytometry-based functional assays (e.g., measure of WASP protein, analysis of gamma chain signaling, assessment of superoxide production), together with the results of XCI studies in the mother, can allow making a diagnosis guiding more complex genomic analyses, for example to search for gene inversions, promoter mutations, and other rare genomic changes. The possibility of false negative exome-sequencing results was reported for WAS [10], X-SCID [95], and chronic granulomatous disease (CGD) [96]. Conversely, for some PIDs, also flow cytometry can yield false negative results, as reported about the expression of SAP protein in XLP [97]. Thus, clinical, immunological, and genomic investigations remain reciprocally necessary for a reliable diagnostic process. Flow cytometry investigations to assess PIDs with immune-dysregulation have been recently reviewed by Cabral-Marques et al. [98].

Case #3 illustrates how primary immune defects may result at a same time in susceptibility to infection and in seemingly unprovoked inflammatory activation. It also highlights how PIDs may be hidden in cases diagnosed with a rheumatological condition. Monogenic autoinflammatory diseases are included in the International Union of Immunological Societies (IUIS) classification of inborn errors of immunity [99]. Apart from the group of pure autoinflammatory disorders, some PIDs can present autoinflammatory symptoms in addition to other signs of immune deficiency. Patients with this kind of disorder can be firstly referred to rheumatologists with the suspicion of a pure autoinflammatory disorder. Table 3 lists some PIDs that can present both infectious and autoinflammatory features.

Inflammatory and autoimmune manifestations can occur in almost all PIDs. However, only a few PIDs display autoinflammatory features with a prevalent involvement of a single cytokine, behaving similarly to typical autoinflammatory conditions and benefiting from specific cytokine blocking treatments. Some exemplary PIDs with autoinflammation are reported in Table 3, highlighting precision therapies, when available.

**Figure 3:**
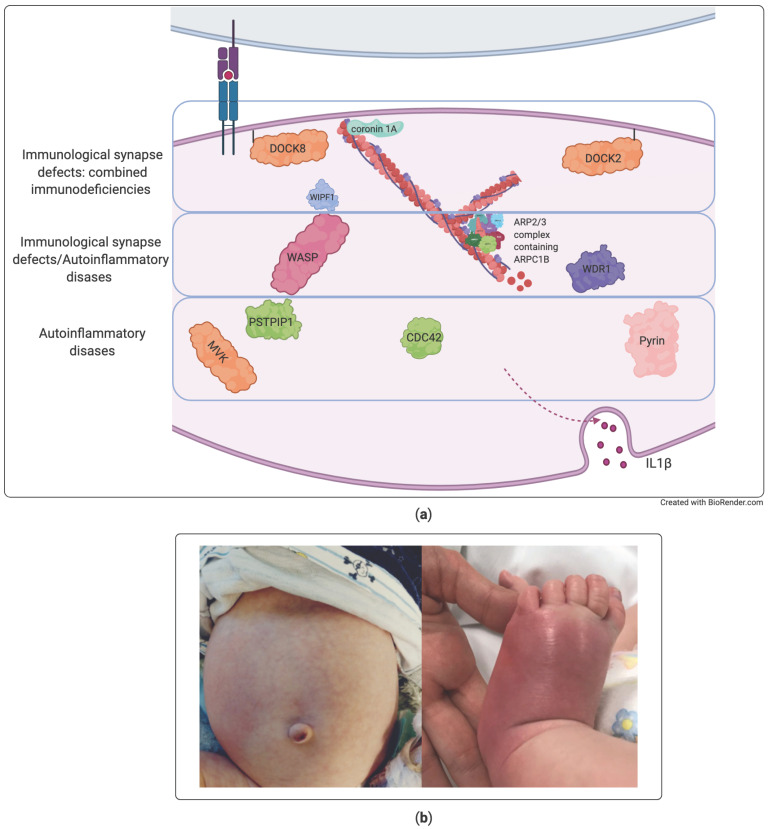
Actinopathies. (**a**) Schematic representation of immune disorders associated with impaired actin cytoskeleton regulation, affecting the immune synapsis and lymphocyte activation, the regulation of IL-1 autoinflammation, or both. (**b**) Autoinflammatory manifestations responsive to IL-1 inhibition in a case of Wiskott–Aldrich syndrome.

Of particular interest is the newly described group of actinopathies [100,101,102], in which the disturbed homeostasis the cytoskeleton can reflect both on defective formation of immune synapsis and on autoinflammatory response. Actinopathies encompass a wide spectrum of diseases ranging from severe immune deficiencies due to defective immune synapsis at one end to autoinflammatory disorders associated with an increased release of IL-1 at the other end (Figure 3). On one hand, an impaired formation of the immune synapse reflects on reduced cooperation between immune cells and defective lymphocyte activation and cytotoxic function, leading to more or less severe combined immunodeficiency, as in DOCK2 [103], DOCK8 [104,105], Coronin 1, and WIP deficiencies. On the other hand, defective dynamics of actin polymerization leads to abnormal activation of pyrin inflammasome and autoinflammation, as in PAPA syndrome [106,107,108], Familial Mediterranean Fever [108,109,110], Hyper-IgD syndrome [109], and CDC42 deficiency [111,112]. Thus, actinopathies may predominantly present with phenotypes related to combined immunodeficiency or to autoinflammation, but some disorders may present with mixed phenotypes, as in the case of Wiskott–Aldrich syndrome [10,113], ARPC1B deficiency [114], or WDR1 deficiency (in which, however a defective neutrophil function is associated with the dysregulation of IL-18 instead of IL-1 [115,116]).

In these disorders, it could be difficult to distinguish infective from autoinflammatory manifestations. In some cases, the ex-juvantibus response to IL-1 inhibition with anakinra has proved useful by providing indirect evidence of the autoinflammatory nature of clinical manifestations such as skin rashes and unexplained fever [10,111].

Of note, some patients with PID associated with autoinflammation may be initially referred to rheumatologists for complaints supportive of SLE or Behçet disease (as in the case of STAT1 GOF, A20 haploinsufficiency syndromes, WDR1 deficiency), and infective symptoms may be misinterpreted as a complication of immunosuppressive treatments [117].

Case #4 presentation serves to illustrate the importance of lymphoproliferative features in raising the suspicion of a PID. APDS has been only recently characterized as a specific PID. Since it may present with variable clinical pictures, subjects with APDS in the past could be classified as having common variable immunodeficiency (CVID), Hyper-IgM syndrome, or combined immunodeficiency (CID). Accordingly, the treatment for patients included prophylactic antibiotics, immunoglobulin replacement, and in some cases, hematopoietic stem-cell transplantation (HSCT). Many other diseases (e.g., CTLA4- Cytotoxic T-Lymphocyte Antigen 4- deficiency, LRBA- LPS responsive beige-like anchor protein- deficiency, CD40/CD40L deficiency), prior to the discovery of their monogenic cause, were classified as CVID, which is a heterogeneous group (the term “variable” refers to this heterogeneity) of disorders characterized by predominant antibody deficiency not due to other well-defined PIDs. More recently, widened awareness of PID together with the availability of NGS techniques led to the discovery of more and more genes underpinning PIDs [123] and to the characterization of new pathogenic mechanisms. This allowed better clustering of pathogenetic pathways and phenotype combinations (endotype) in already established PID, in some cases paving the way to the development of targeted treatments.

Therefore, when a new mutation in a specific disease-related gene is identified, this new clinical entity should be considered as independent disease, especially when the identification of the causative gene gives us a therapeutic target for precision therapies that can be implemented in addition to, or even before, conventional prophylactic treatments with antimicrobials and immunoglobulins [11].

Of particular interest are two groups of disorders, respectively characterized by hyperactivation of the PI3K–AKT–mTOR pathway, as autoimmune lymphoproliferative syndrome (ALPS), APDS, immuno-TORpathies [124,125], and by defective function of regulatory T cell functions, such as immunodysregulation polyendocrinopathy enteropathy X-linked (IPEX), CTLA4 deficiency, and LRBA deficiency. Both groups of diseases are associated with lymphoproliferation, often with chronic enlargement of spleen and liver in addition to peripheral lymph nodes. However, the clinical phenotype and the assessment of biomarkers can help distinguish these conditions even before performing NGS. For example, high serum vitamin B12 is typically elevated in ALPS; brain, lung and gut lesions may characterize CTL4 and LRBA deficiency, and chronic EBV infections are more typical of disorders such as X-linked immunodeficiency with magnesium defect (XMEN, already mentioned in Table 2).

Although only two cases have been described so far, enlargement of lingual tonsil might be a sign of lymphoproliferation supportive of APDS. Of note, lymphoproliferative and autoimmune symptoms in ALPS and APDS may respond to treatment with sirolimus even at a lower than usual dosage [126,127].

More interestingly, other treatments can even rescue the defective immune development, as in the case of PI3K inhibitors in APDS syndrome [128,129,130], or correct autoimmune features related to abnormal immune activation, as in the case of CTLA4 or LRBA deficiencies, which can strongly benefit from a therapy with abatacept, surrogating the defective regulatory function of CTLA4 [131,132].

Most of these lymphoproliferative diseases also constitute a form of susceptibility to Epstein–Barr virus (EBV) infection, and in some cases, lymphoproliferation may be evident only after such infection.

EBV infects about 90% of the population by 30 years of age, being clinically manifested during the primary infection and remaining latent in B or T/NK lymphocytes. It is known that it induces lymphoproliferation, especially of B lymphocytes, but also T and NK cells. Subjects with PID, especially those with defective T cell immunity or those with minor cytotoxic defects accounting for specific susceptibility to EBV, are less able to control the infection and have a high risk of developing EBV-positive lymphocyte proliferation diseases (LPDs) and leukemia/lymphomas, or they may show clinical manifestations consistent with chronic active EBV disease (CAEBV, XMEN) [133,134].

**Figure 4:**
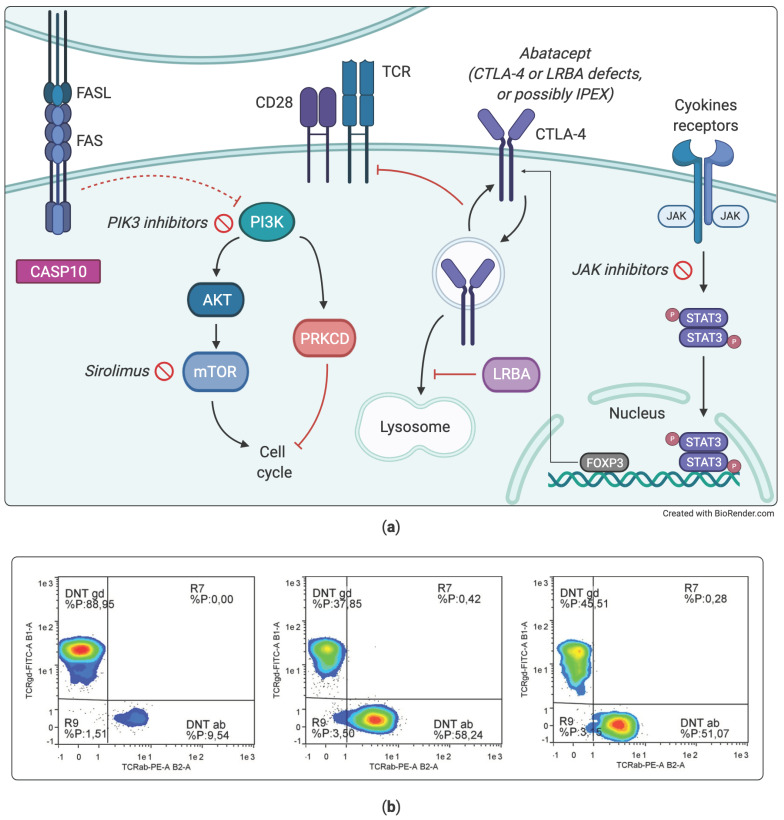
Examples of pathogenic mechanisms in immunodeficiencies with lymphoproliferation (**a**) PI3K–AKT–mTOR pathway from APDS to ALPS and immuno-TORpathies. (**b**) Representative flow cytometry dot plot of double negative T cells expressing alpha/beta or gamma/delta T cell receptor (supplementary materials). Percentage values shown in the graphs are calculated on CD4-CD8- T cells; percentage values of DNT alpha/beta calculated on CD3+ population are 1,4-15, 7-5. From the left to the right: healthy control, ALPS syndrome and LRBA deficiency.

We do not yet know if precision treatments available for some of these PIDs with lymphoproliferation may impact on lymphoma risk. As we previously wrote [127], the experience of sirolimus in kidney transplantation suggests that this medication affects cancer risk less than other immunosuppressive treatments or even reduces it. Based on these data, we thought that mTOR inhibitors (everolimus/sirolimus) prescribed in immunological diseases with a hyperactivated PI3K–AKT–mTOR pathway (e.g., ALPS, APDS, APDS-like) might also be effective in reducing the risk of developing immune malignancies, even if there is still no clinical evidence about this (Figure 4). Table 4 shows a group of immunodeficiency with lymphoproliferation treatable with precision therapies. In general, lymphoproliferation is associated with increased risk of developing lymphoma. A more complete list of PIDs associated with immune dysregulation can be found in the updated IUIS classification [99].

Case #5 is an example of PIDs associated with susceptibility to specific infections. It is worth noting that subjects affected with these PIDs may have a completely healthy life until the encounter of a specific microbe that highlights a single weak point in the system. Indeed, the apparent redundancy of the immune system can be easily understood when we can see how apparently minor immune defects may result in serious consequences. Table 5 summarizes some conditions of specific Mendelian susceptibility to infections. In general, they concern microbes or viruses that evolved effective mechanisms to elude most (but not all) of the host’s immune defenses.

## 4. Conclusions

**Figure 5:**
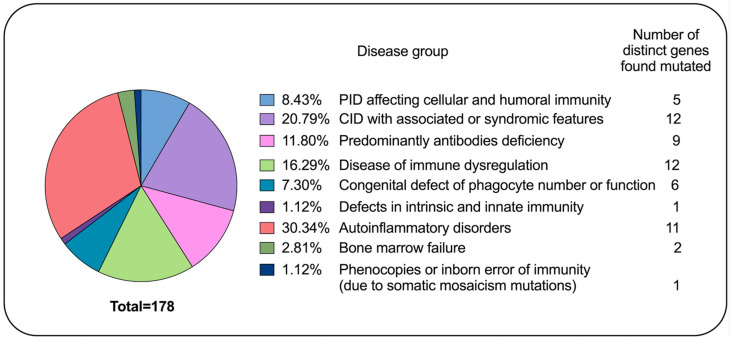
Primary immunodeficiency disease (PID) diagnosis in the last twenty-years in the experience of a pediatric hospital in Italy. The pie chart displays the proportion of subject affecting by distinct inborn error of immunity and the number of diverse genes mutated for each condition.

PIDs include more than 300 genetic disorders that can be classified in 10 categories according to the IUIS [99]. Clinical exome is gaining more and more indication for diagnosing, but the integration with clinical and immunological data is still critical to make a proper and defined diagnosis. In the experience of a pediatric reference hospital in Italy, in the last twenty-five years, we diagnosed 178 subjects with inborn error of immunity due to mutations in about 59 distinct genes, highlighting the high genetic heterogeneity underpinning PID (Figure 5). The five settings described in the present article are meant only for exemplary purposes. In fact, many PIDs can fit into diverse scenarios, and many others cannot fit into any of them. The aim of our presentation is just to highlight the five general messages resumed in Summary Box. Furthermore, we chose to include some PIDs in which a proper diagnosis can allow prescribing precision therapy with a favorable prognostic impact (druggable PIDs). A similar principle is at the basis of the initiative from the Jeffrey Modell Foundation to promote a global genetic sequencing pilot program to identify specific primary immunodeficiency defects to optimize disease management and treatment [94]. Indeed, since the clinical phenotype of “druggable” PIDs may overlap with common rheumatologic or gastroenterological disorders, it is important to increase the awareness of the possible genetic diagnosis among diverse medical specialists.

**Summary Box**

SCIDs can be identified and classified by basic flow cytometry. Deficiency in recent thymic emigrants (RTE) is highly indicative of SCID. Defective thymic output can be also measured by molecular techniques (TREC analysis) in dry blood spots in newborn screening. Positive results from TREC screening require further analysis to obtain a diagnosis, with both flow cytometry techniques and NGS panels.

Several PID have X-recessive inheritance. Immunological assays remain of crucial importance both for driving the diagnosis (as in the case of dihydrorhodamine (DHR) test for CGD or measure of WASP expression in WAS) and for confirming the causative role of variants of uncertain significance (VUS) detected by NGS. X-chromosome inactivation (XCI) analysis in appropriate subsets of cells from heterozygous mothers still has a role in interpreting genetic results. Moreover, the study of XCI is useful also in rare cases of heterozygous females affected because of unbalanced XCI. Other specialist tests can help diagnosis in specific disorders such as dyskeratosis congenita (telomere length analysis), ectodermal dysplasia with immunodeficiency (response to TLRs), and X-SCID (response to IL-2 stimulation).

Several PIDs can present with autoinflammatory phenotype. Distinguishing autoinflammation from infection is a critical issue. Basic flow cytometry and genetics may not be sufficient to recognize the autoinflammatory component in PIDs, whilst the assessment of serum biomarkers and transcriptomic signatures can be greatly useful. Ex-juvantibus response to selective anti-cytokine treatment can also give valuable help.

Lymphoproliferation is also a feature shared among various PIDs. Increased Double Negative T cells are typical of ALPS and can be found also in other ALPS-like conditions. The assessment of antigens related to immune regulation, such as FOXP3 and CTLA4, is also of some help, but NGS panels are increasingly adopted in subjects with immunodeficiency and lymphoproliferation. However, VUS are often encountered, which can be of challenging interpretation, especially when associated with protein gain-of-function (GOF), as in the case of activated PI3K kinase syndrome or STAT3 GOF.

Mendelian susceptibility to specific infections, such as candida, EBV, herpes viruses, and mycobacteria, is not easily detected by common immunological investigations. NGS is often used to address the diagnosis, but specialistic immune assays may be required to interpret the pathogenic role of VUS.

## Figures and Tables

**Table 1:** Diseases with low RTE/TREC.

Primary Immune Defects		
SCID	T-B-NK+ T-B+NK- T-B-NK- Other combinations	Assessment of STAT phosphorylation can help subclassification [36,37]. Subjects with engraftment of maternal lymphocytes may have normal T cell count but have absent RTE [5].
Combined ID	CHH Pediatric onset CVIDs Del 22q11.2 syndrome and other immunodeficiencies with defective thymic development [38]. Other CID [39]	CVIDs with severe lymphopenia and/or severe reduction of naïve T cells should be classified as CID [40,41] Pediatric onset CVIDs have usually low RTE [42], with the exception of NFKB2 deficiency that may display increased RTE [43,44]. Assessment of associated clinical features can help the identification of specific syndromes such as CHH [45], 22q11.2 syndrome [46,47,48], and APDS (*PIK3CD* and *PIK3R1* gene) [49].
**Secondary Immune Defects** [50]		
Preterm infant		Specific reference values for naive T and B cells have to be considered in preterm infants [51,52]
Neonatal thymectomy		Reduced RTE [53,54]
HIV infection		Increase of RTE if HAART started < 6 months from infection [55]
HSCT		Increase of RTE if reconstitution from pre-thymic precursors [56,57,58]
Drugs		Cytostatic drugs, cyclosporine [59]
Elderly people		Immunosenescence [60]

Note: SCID: severe combined immunodeficiency; RTE: recent thymic emigrants; CHH: cartilage-hair hypoplasia; CVID: common variable immunodeficiency; CID: combined immunodeficiency; APDS: activated phosphoinositide 3-kinase δ syndrome; HIV: human immunodeficiency virus; HAART: highly active antiretroviral therapy; HSCT: hematopoietic stem-cell transplantation; TREC: T cell receptor excision circle.

**Table 2:** Functional assay to address suspicion or to assess relevance of genetic variants of unknown significance.

Disease (Gene)	XCI Skewed in	Routine Lab and Immunophenotype	Disease in Hz Females with Skewed Inactivation	Functional Assays
X-SCID (*IL2RG*)	T cells and NK [61]	B+ T- NK-	Not described	Proliferation; response to IL-2
WAS (*WAS*)	WBC [64]	Leukopenia; thrombocytopenia with small platelets	From XLT to WAS (due to skewed XCI [65,66,67] or uniparental isodisomy 6 [68])	WASP expression (cytometry); proliferation
CGD (*CYBB*)	No effect	Leukocytosis	From lupus discoid and stomatitis to CGD [69,70,71]	DHR or NBT test
XLA (*BTK*)	B cells [72]	Low B cells	[73]	BTK expression
IPEX (*FOXP3*)	Tregs [74]	High activation markers	Autoimmune disorders possibly associated with carrier status [75]	Measure of Tregs numbers; measure of TSDR [76]
NEMO deficiency (*IKBKG*)	WBC [77]	Non-specific	Icontinentia pigmenti [78,79]	Response to TLRs [78,80]
Dyskeratosis congenita XLR (*DKC1*)	WBC [81]	Non-specific	[81,82]	Telomere flow-FISH [83]
XLP2 (*XIAP*)	No effect	Non-specific	HLH [84], Crohn’s Disease [85]	Measure of XIAP expression [86]
XLP1 (*SH2D1A*)	No effect	Non-specific	Dysgammaglobulinemia [87,88]	Measure of SAP expression [86]
HIGM1 (*CD40LG*)	No effect	Reduced switched memory B cells	XCI [89,90]	Measure of CD40LG on activated lymphocytes
XMEN (*MAGT1*)	Leukocytes [91]	Reduced RTE	Not described	Glycosylation defect [92]

Note: XCI: X-chromosome inactivation; Hz: heterozygous; X-SCID: X-linked severe combined immunodeficiency; WAS: Wiskott–Aldrich syndrome; WBC: white blood cells; XLT: X-linked thrombocytopenia; CGD: chronic granulomatous disease; DHR: dihydrorhodamine; NBT: nitroblue tetrazolium; XLA: X-linked agammaglobulinemia; IPEX: immunodysregulation polyendocrinopathy enteropathy X-linked; TSDR: Treg-specific demethylated region; XLR: X-linked recessive; FISH: fluorescence in situ hybridization; XLP: lymphoproliferative syndrome, X-linked; HLH: hemophagocytic lymphohistiocytosis; HIGM1: X-linked hyper-IgM syndrome; XMEN: X-linked immunodeficiency with magnesium defect, Epstein-Barr virus infection, and neoplasia; RTE: recent thymic emigrants.

**Table 3:** Immunodeficiencies with autoinflammation.

Disease	Gene	Predominant Cytokine	Precision Therapies for the Autoinflammatory Component
STAT1 GOF syndrome	*STAT1* GOF	Interferons	JAK inhibitors
Wiskott–Aldrich syndrome	*WAS* [10]	IL-1 *	Anakinra
Immunodeficiency 71 with inflammatory disease and congenital thrombocytopenia	*ARPC1B* [114]		
Immunodeficiency with dyshematopoiesis, inflammation, and HLH	*CDC42* [112]		
Autoinflammation, antibody deficiency, and immune dysregulation syndrome (APLAID)	*PLGC2* GOF [118]	IL-1	Anakinra
Autoinflammatory periodic fever, immunodeficiency, and thrombocytopenia (PFIT)	*WDR1* [116,119,120]	IL-18	Partial response to anakinra
Immunodeficiency 72 with autoinflammation	*NCKAP1L* [121]	IL-18, IFN-g	Not defined
A20 haploinsufficiency	*TNFAIP3* [122]	TNF-alpha	Anti-TNF

* Disturbance of the actin cytoskeleton in these three disorders, also referred to as pertaining to the new group of actinopathies, is associated with IL-1 mediated autoinflammation responsive to anakinra [101]. Note: HLH: hemophagocytic lymphohistiocytosis.

**Table 4:** Immunodeficiencies with lymphoproliferation.

Disease	Genes	Immuno-Phenotype	Lympho-Proliferation	Autoimmune Cytopenia	Enteropathy	Infections	Inheritance	Precision Therapy
ALPS [135]	*FAS* *FASLG* *CASP10* *PRKCD*	DNT	++	++ SLE-like features	---	EBV-induced lymphoproliferation	AD, AR	Sirolimus [136,137] [127,138,139]
IPEX	*FOXP3*	Activated lymphocytes	+	++	+++	---	XLR	Sirolimus [140,141]
APDS	*PIK3CD* *PIK3R1*	Senescent CD8 CD57+ T cells; reduced switched memory B cells	++	+	+	+	AD	Sirolimus, PI3K inhibitors
STAT3 GOF	*STAT3*	DNT	++	++	---	---	AD	JAK inhibitors
CTLA4 deficiency	*CTLA4*	Sometimes, reduced CTLA4 expression	++, risk of lymphoma	++	++	+	AD	Abatacept, sirolimus
LRBA deficiency	*LRBA*	Reduced LRBA expression; reduced CTLA4 expression	++, risk of lymphoma	+	++	+	AR	Abatacept, sirolimus

Note: ALPS: autoimmune lymphoproliferative syndrome; DNT: double negative T cells; SLE: systemic lupus erythematosus; EBV: Epstein–Barr virus; AD: autosomal dominant; AR: autosomal recessive; IPEX: immunodysregulation polyendocrinopathy enteropathy X-linked; XLR: X-linked recessive; APDS: activated phosphoinositide 3-kinase δ syndrome; CTLA4: Cytotoxic T-Lymphocyte Antigen 4; LRBA: LPS responsive beige-like anchor protein; ---: not present; +: mild expression; ++: moderate expression; +++: severe expression.

**Table 5:** Susceptibility to severe course from specific microbial infections.

Selective Susceptibility	Main Involved Pathway	Phenotypes	Reviewed	Precision Therapies
Candida	Defective Th17 function, defective sensing of candida	Mucocutaneous candidiasis, hyper-IgE syndrome, recurrent oral ulcerations, candidiasis with SLE-like overlap (STAT1 GOF), APECED (autoimmune polyendocrinopathy-candidiasis-ectodermal dystrophy)	[142]	JAK inhibitors in STAT1 GOF [143] G-CSF in CARD9 deficiency [144] Only anti-candida prophylaxis in APECED
EBV	Cytotoxic lymphocyte and NK function; CD8 T cell/APC synapse formation; CD8 T cell priming; EBV specific CD8 T cell function	CAEBV, B lymphoproliferative disorder, HLH, persistent lymphoproliferation and hematologic autoimmunity (as in ALPS and APDS syndromes)	[145,146]	Anti-CD20 therapy helps clearing the viral reservoir; donor lymphocyte infusions with whole peripheral blood or EBV-specific T cell lines; EBER cytometry to characterize the infected cells [147]
Warts and HPV related disorders	Deficiency in CD4 function; APC functions; keratinocyte innate immunity	Epidermodysplasia verruciformis, non-melanoma skin cancers, cutaneous warts, anogenital lesions	[148]	Specific inhibitors for CXCR4 for WHIM (Warts, Hypogammaglobulinemia, Infections, and Myelokathexis).
HSV	Sensing of viral components and Interferon cascade	Encephalitis (especially if recurrent), disseminated herpes virus infection	[149,150,151,152,153]	Antiviral therapy and prevention of relapses
Mycobacteria	Pathway of sensing mycobacterial PAMPS to the production of gamma interferon in dendritic cells, phagocytes, lymphocytes and natural killer cells	BCGitis, Disseminated atypical mycobacteriosis,	[154]	JAK inhibitors in STAT1 GOF Recombinant IFN-g in patients with IL12 cascade deficiency [155] Recognize CGD and NEMO deficiency

Note: Several other PIDs may be associated with any of these specific infections; however, in these cases, the susceptibility is not so selective. No mention is made to defects with broader susceptibility to infections. SLE: systemic lupus erythematosus; APECED: autoimmune polyendocrinopathy–candidiasis–ectodermal dystrophy; G-CSF: granulocyte-colony stimulating factor; EBV: Epstein–Barr virus; APC: antigen-presenting cells; CAEBV: chronic active EBV infection; HLH: hemophagocytic lymphohistiocytosis; ALPS: autoimmune lymphoproliferative syndrome; APDS: activated phosphoinositide 3-kinase δ syndrome; HPV: human papilloma virus; WHIM: Warts, Hypogammaglobulinemia, Infections, and Myelokathexis; HSV: herpes simplex virus; PAMPS: pathogen-associated molecular patterns; CGD: chronic granulomatous disease.

## Data Availability

The data presented in this study are available on request from the corresponding author. The data are not publicly available due to preserve patients’ privacy.

## Supplementary Materials

The following are available online at https://www.mdpi.com/2075-4418/11/3/532/s1 which describe in more details laboratory methods that have been used.
