# Supplementary material for: Immunity and Genetics at the Revolving Doors of Diagnostics in Primary Immunodeficiencies
Source: Diagnostics (Basel). 2021 Mar 16;11(3):532. doi: 10.3390/diagnostics11030532 (PMC8002250; doi:10.3390/diagnostics11030532)
Supplement: Supplementary file 1 [file diagnostics-11-00532-s001.pdf]

Supplementary material and methods

X-chromosome inactivation (XCI) assay

Genomic DNA (gDNA) was extracted from EDTA-anticoagulant blood using an EZ1 DNA Blood kit (QIAGEN GmbH, Hilden, Germany). A total of 300 ng gDNA was digested with *HpaII*, methylation-sensitive restriction enzyme, and *RsaI* (New England Biolabs, Ipswich, Massachusetts, USA) in a final volume of 50µl for 24 h at 37°C. 50 ng of digested and undigested samples were amplified using PCR primers, one of which labeled with FAM (Eurofins Genomics, Luxembourg). PCR primers amplified the region of the human androgen receptor (*AR*) gene, localized on Xq12 and characterized by a polymorphic CAG repeats and two *Hpa*II sites. Only the methylated (inactivated) allele was amplified. The labeled PCR products were analyzed by an automated sequencer (ABI 3500DX; Applied Biosystems, Waltham, MA, USA) and GeneMapper software (v.4.1; Applied Biosystems, Waltham, MA, USA) was used for quantifying the area under the curve. The percentage of inactivation is considered skewed for values greater than 80% [1].

NGS panel and sequencing

A custom-made panel for targeted interest genes can be created using Ion AmpliSeq Designer (Thermo Fisher, Waltham, MA, USA). DNA libraries were generated using Ion Ampliseq Library kit 2.0 (Thermo Fisher, Waltham, MA, USA), purified with magnetic bead technology Agencourt AMPure XP (Beckman Coulter, Brea, CA, USA) and quantified with KAPA Library Quantification Kits (Roche, Basel, Switzerland) according to manufacturer's protocols. The sequencing step was performed on the Ion TorrentTM PGM platform (Thermo Fisher, Waltham, MA, USA). Torrent SuiteTM software v5.12 was used to analyze the signal processing and the output file was further annotated using wANNOVAR free software (http://wannovar.wglab.org/).

Criteria used for filtering variants are the following: minor allele frequency reported in gnomAD browser (<0.02 if recessive inheritance model or <0.001 if dominant inheritance model), type of mutation (non-synonymous, nonsense, frameshift, splicing about 10 nucleotides from the splice site), damaging prediction (at least two of the four *in silico* prediction tools Polyphen-2, SIFT, Mutation Taster and Mutation Assessor; a high CADD score (>15) and GERP score as a measure of the conservation of the genomic position) [2-7] and phenotype correlation (Human Gene Mutation Database professional to define association with mutation and specific phenotype).

Potential causative variants were validated by Sanger Sequencing (proband and parents). Polymerase chain reaction amplification was performed using the KAPA 2G Fast Hot Start Readymix (RESNOVA, Roma, Italy), and subsequently processed and sequenced by Eurofins Genomics.

Dihydrorhodamine assay (DHR)

To evaluate the capacity of neutrophils to product oxygen reactants (ROS) a commercial kit was used (FagoFlowEx, EXBIO, Vestec, Czech). Briefly, 50 µL of heparinized peripheral blood sample was treated with phorbol 12-myristate 13-acetate (PMA) to induce the production of ROS by the NADPH oxidase enzyme activity. The release of ROS was then revealed thanks to a molecule, dihydrorodamine 123 (DHR123), which was converted to fluorescent rhodamine 123. The fluorescence signal was read by means of a cytometer, MACSQuant Analyzer 10, and analyzed with FlowLogic software.

Lymphocytes subpopulations

Multicolor immunophenotyping was performed on 100 µL of heparinized peripheral blood samples in order to evaluate different lymphocyte subsets, including recent thymic emigrants, transitional B cells, naïve B cells, IgM memory B cells and switched memory B cells, double negative T cells and senescent CD8 T cells. After 20′ of incubation with specific antibodies mixtures, samples were treated with a solution to lyse red blood cells and fix lymphocytes (FACS lysing solution, BD, Franklin Lakes, NJ, USA).

Four antibodies panels were used:

- Panel for RTE analysis contained the following antibodies: anti-CD45, anti-CD19, anti-CD4, anti-CD8 (all from BD, Franklin Lakes, NJ, USA), anti-CD3, anti-CD45RA, anti-CD31, anti-CD16 and anti-CD56 (all from Miltenyi Biotec, Bergisch Gladbach, Germany).
- Panel for B cells analysis contained the following antibodies: anti-CD45, anti-CD19, anti-CD38 (all from BD, Franklin Lakes, NJ, USA), anti-CD27, anti-IgM, anti-IgG, anti-CD21, anti-CD10 and anti-IgD (all from Miltenyi Biotec, Bergisch Gladbach, Germany).
- Panel for double negative T cells contained the following antibodies: anti-CD45, anti-CD8, anti-CD4 (all from BD, Franklin Lakes, NJ, USA), anti-CD3, anti-TCRgd, anti-TCRab, anti-B220 and anti-CD95 (all from Miltenyi Biotec, Bergisch Gladbach, Germany).
- Panel for senescent CD8 T cells contained the following antibodies: anti-CD45, anti-CD8 (both from BD, Franklin Lakes, NJ, USA), anti-CD4, anti-CD3, anti-CD57, anti-CD45RA and anti-CD27 (all from Miltenyi Biotec, Bergisch Gladbach, Germany).

Samples were acquired with MACSQuant Analyzer 10 and analyzed with FlowLogic software.

WASP expression

100 μL of heparinized peripheral blood samples obtained from the patient and from healthy control were stained with anti-CD45 antibody (BD, Franklin Lakes, NJ, USA). After 20' of incubation cells were fixed and permeabilized, respectively, with Fixation Medium A and Fixation Medium B (ADG, Wien, Austria). To perform intracellular staining of WAS protein, a monoclonal antibody anti-WASP (BD, Franklin Lakes, NJ, USA) followed by a secondary anti-mouse IgG2a antibody (Biolegend, San Diego, CA, USA) were used.

Samples were acquired with MACSQuant Analyzer 10 and analyzed with FlowLogic software.

Interferon signature and interferon score

Peripheral blood was collected in PAXgene Blood RNA Tubes (PreAnalytiX, Hombrechtikon, Switzerland), extracted with PAXgene Blood RNA Kit (PreAnalytiX, Hombrechtikon, Switzerland) following the manufacturer's instructions, quantified with NanoDrop 2000 Spectrophotometer (Thermo Fisher, Waltham, MA, USA) and retro-transcribed using SensiFAST cDNA Synthesis Kit (Bioline, London, UK).

The expression of six interferon stimulated genes (*IFI27*, *IFI44L*, *IFIT1*, *ISG15*, *RSAD2* and *SIGLEC1*) was measured by qPCR with AB 7500 Real Time PCR System (Applied Biosystems, Waltham, MA, USA), TaqMan Gene Expression Master Mix (Applied Biosystems, Waltham, MA, USA) and UPL Probes (Roche, Basel, Switzerland). Using AB 7500 Real Time PCR software, each target quantity was normalized with the expression level of *HPRT1* and *G6PD*, and the relative quantification was calculated relating to a "calibrator" sample (mix of ten healthy controls) using the $2^{-\Delta\Delta Ct}$ method. The median fold change of the six target genes provides the interferon score, which represents the intensity of interferon signature [8].

References

1. Vousooghi, N.; Shirazi, M.S.; Goodarzi, A.; Abharian, P.H.; Zarrindast, M.R. X Chromosome Inactivation in Opioid Addicted Women. *Basic Clin Neurosci* **2015**, *6*, 179-184.
2. Adzhubei, I.A.; Schmidt, S.; Peshkin, L.; Ramensky, V.E.; Gerasimova, A.; Bork, P.; Kondrashov, A.S.; Sunyaev, S.R. A method and server for predicting damaging missense mutations. *Nat Methods* **2010**, *7*, 248-249, doi:10.1038/nmeth0410-248.
3. Kumar, P.; Henikoff, S.; Ng, P.C. Predicting the effects of coding non-synonymous variants on protein function using the SIFT algorithm. *Nat Protoc* **2009**, *4*, 1073-1081, doi:10.1038/nprot.2009.86.
4. Chun, S.; Fay, J.C. Identification of deleterious mutations within three human genomes. *Genome Res* **2009**, *19*, 1553-1561, doi:10.1101/gr.092619.109.
5. Schwarz, J.M.; Rödelsperger, C.; Schuelke, M.; Seelow, D. MutationTaster evaluates disease-causing potential of sequence alterations. *Nat Methods* **2010**, *7*, 575-576, doi:10.1038/nmeth0810-575.
6. Kircher, M.; Witten, D.M.; Jain, P.; O'Roak, B.J.; Cooper, G.M.; Shendure, J. A general framework for estimating the relative pathogenicity of human genetic variants. *Nat Genet* **2014**, *46*, 310-315, doi:10.1038/ng.2892.
7. Cooper, G.M.; Goode, D.L.; Ng, S.B.; Sidow, A.; Bamshad, M.J.; Shendure, J.; Nickerson, D.A. Single-nucleotide evolutionary constraint scores highlight disease-causing mutations. *Nat Methods* **2010**, *7*, 250-251, doi:10.1038/nmeth0410-250.
8. Volpi, S.; Insalaco, A.; Caorsi, R.; Santori, E.; Messia, V.; Sacco, O.; Terheggen-Lagro, S.; Cardinale, F.; Scarselli, A.; Pastorino, C.; et al. Efficacy and Adverse Events During Janus Kinase Inhibitor Treatment of SAVI Syndrome. *J Clin Immunol* **2019**, *39*, 476-485, doi:10.1007/s10875-019-00645-0.
